# Dr. Herbert M. Burritt

**Published:** 1917-12

**Authors:** 


**Dr. Herbert M. Burritt,** Buffalo 1901, of Holton, Monroe Co., N. Y., died at Clarkson, N. Y., Oct. 12, aged 45.

